# Informal Health Provider and Practical Approach to Lung Health interventions to improve the detection of chronic airways disease and tuberculosis at primary care level in Malawi: study protocol for a randomised controlled trial

**DOI:** 10.1186/s13063-015-1068-4

**Published:** 2015-12-17

**Authors:** Hastings T Banda, Kevin Mortimer, George AF Bello, Grace B Mbera, Ireen Namakhoma, Rachael Thomson, Moffat J Nyirenda, Brian Faragher, Jason Madan, Rasmus Malmborg, Berthe Stenberg, James Mpunga, Beatrice Mwagomba, Elvis Gama, Katherine Piddock, Stephen B Squire

**Affiliations:** Research for Equity and Community Health Trust, ᅟ, Malawi; Liverpool School of Tropical Medicine, Liverpool, UK; Ministry of Health, 30377, Capital City, Lilongwe 3, Malawi; College of Medicine, Mahatma Gandhi, Blantyre, Malawi; LHL International, Grensen 3, 0159, Oslo, Norway; Warwick University, Coventry, CV4 7AL UK

**Keywords:** Tuberculosis, Chronic airways disease, Non-communicable disease, Practical Approach to Lung Health

## Abstract

**Background:**

In developing countries like Malawi, further investigation is rare after patients with chronic cough test negative for tuberculosis. Chronic airways disease has presentations that overlap with tuberculosis. However, chronic airways disease is often unrecognised due to a lack of diagnostic services. Within developing countries, referral systems at primary health care level are weak and patients turn to unskilled informal health providers to seek health care. Delayed diagnosis and treatment of these diseases facilitates increased severity and tuberculosis transmission.

The World Health Organisation developed the Practical Approach to Lung Health strategy which has been shown to improve the management of both tuberculosis and chronic airways disease. The guidelines address the need for integrated guidelines for tuberculosis and chronic airways disease. Engaging with informal health providers has been shown to be effective in improving health services uptake. However, it is not known whether engaging community informal health providers would have a positive impact in the implementation of the Practical Approach to Lung Health strategy. We will use a cluster randomised controlled trial to determine the effect of using the two interventions to improve case detection and treatment of patients with tuberculosis and chronic airways disease.

**Methods:**

A three-arm cluster randomised trial design will be used. A primary health centre catchment population will form a cluster, which will be randomly allocated to one of the arms. The first arm personnel will receive the Practical Approach to Lung Health strategy intervention. In addition to this strategy, the second arm personnel will receive training of informal health providers. The third arm is the control. The effect of interventions will be evaluated by community surveys. Data regarding the diagnosis and management of chronic cough will be gathered from primary health centres.

**Discussion:**

This trial seeks to determine the effect of Informal Health Provider and Practical Approach to Lung Health interventions on the detection and management of chronic airways disease and tuberculosis at primary care level in Malawi.

**Trial registration:**

The unique identification number for the registry is PACTR201411000910192 – 21 November 2014

**Electronic supplementary material:**

The online version of this article (doi:10.1186/s13063-015-1068-4) contains supplementary material, which is available to authorized users.

## Background

Among non-communicable diseases (NCDs), respiratory diseases represent a major burden of disease, in particular chronic airways disease (CAD). CAD includes asthma, chronic obstructive pulmonary disease (COPD) and bronchiectasis. Risk factors for these conditions include tobacco-smoking, indoor air pollution from biomass fuels and lower respiratory tract infections.

Chronic or persistent coughing is a frequent clinical presenting feature of both CAD and tuberculosis (TB). Chronic cough is a distressing symptom and a common reason for people to seek health services [1, 2]. Findings from studies conducted in Malawi show that patients presenting with cough exert a huge burden on out-patient department (OPD) services [3], and yet there are many challenges to providing effective care for patients with respiratory symptoms in the OPD [4].

### Chronic airways diseases

COPD is characterised by airflow limitation that is not fully reversible but nonetheless treatable. The World Health Organisation (WHO) estimates that 65 million people have moderate to severe COPD with 5 % of all (3 million) global deaths being attributed to COPD [5]. About 90 % of these COPD deaths occur in low-income and middle-income countries. At the other end of the spectrum, asthma, a chronic inflammatory disorder of the airways, is characterised by episodes of reversible breathing problems (that may be life-threatening) due to airway narrowing and obstruction. These episodes are characterised by coughing, wheezing, chest tightness and shortness of breath. The frequency and severity of these episodes can have a marked impact on the livelihoods and the well-being of those affected [6]. Bronchiectasis is a severe, chronic infection of the lung associated with permanent abnormal dilatation of bronchi [7]. As a result mucus clearance is impaired, leading to accumulation of secretions, bacterial overgrowth and recurrent infections.

Regular therapy of CAD with the rational use of inhaled corticosteroids and bronchodilators can reduce the frequency and severity of exacerbations. To date, inhaled corticosteroids and bronchodilators have not been consistently available for use anywhere in Malawi’s health system, and particularly not at primary care level. Because it is difficult to diagnose CAD without appropriate equipment, unnecessary prescriptions of antibiotics are made for treatment. Where available, inhalers are not used effectively because the patients lack adequate education in their proper use. Some patients are treated empirically for TB (sometimes repeatedly) despite negative sputum smears because of a lack of tools and guidelines for further screening for CAD.

### Tuberculosis

TB is the world’s leading infectious cause of death in adults; approximately 1.4 million deaths out of the 8 million annual TB cases occur per year. TB predominantly affects the respiratory system and chronic or persistent coughing is a cardinal symptom. The Stop TB strategy relies heavily on identifying and treating TB cases from among the large group of patients who seek health care because of persistent coughing. However, TB case detection remains a challenge in most countries [8] and especially across sub-Saharan Africa (SSA). Therefore, adult individuals with chronic respiratory symptoms are potential TB cases and pose a relatively high risk of onward transmission of TB. The WHO estimates that Malawi is detecting only 50 % [9] of infectious TB cases, which is below the global target of 70 % [9].

### The relationship between non-communicable chronic respiratory disease and TB

In developing countries with a high prevalence of TB, only between 10 and 20 % of patients presenting in primary care with persistent coughing will actually have TB. The majority of the remaining 80 to 90 % of patients will be suffering from a wide range of non-communicable chronic respiratory diseases including CAD. A proportion of patients who have TB will also have underlying non-communicable chronic respiratory disease. It is now increasingly recognised that chronic respiratory diseases and TB share risk factors including smoking and low socioeconomic status. It is also recognised that a proportion of patients who have not been treated effectively for TB will develop chronic respiratory diseases, such as bronchiectasis and fibrosis, as a result of the tissue destruction and subsequent scarring [10].

### The impact of chronic airways disease and TB on disability and quality of life

Severe CAD and TB exert disabling effects that reduce quality of life and livelihood opportunities for the affected individuals [11]. It is not known, however, what proportion of patients with COPD are so severely affected as to fulfil international criteria for disability, measured by level and type of activity limitation [12]. People with disabilities face particular challenges in accessing social and health services and have higher risks of disease, including TB infection [13]. These challenges, which include stigma and discrimination, may cause patients with disabilities to fail to access health services, remain in a morbid state, and eventually be caught in a damaging cycle of disability, poverty and ill-health.

### The need for integrating approaches to CAD and TB case detection and treatment

Despite the relationship between CAD and TB, health systems in most developing countries have mainly focused on the detection and cure of infectious diseases including TB. There has been less emphasis on the diagnosis and life-long management of CAD. As a result, patients with chronic cough who do not end up with a TB diagnosis are often frustrated because they are not given a diagnosis, education about their condition or treatment. Given the relationship between TB and CAD, integrated management could reduce the proportion of patients with chronic cough without a diagnosis, reduce costs and reduce multiple visits to health facilities for diagnosis.

For this reason, within the Stop TB strategy framework, WHO and the International Union Against Tuberculosis and Lung Disease (IUATLD) developed the Practical Approach to Lung Health (PAL) strategy. PAL is a patient-centred approach to the diagnosis and treatment of common respiratory illnesses in health care settings other than health centres at primary health care level. Specifically, the PAL strategy aims to improve the quality of respiratory disease management by promoting a symptom-based and integrated approach to diagnosis and treatment. The aim is to ensure standardised service delivery by the implementation of standardised clinical and diagnostic guidelines. The PAL strategy focuses on TB, pneumonia, asthma and COPD.

In Syria and Jordan, where the PAL strategy has been adopted, they have seen improvements in referrals, sputum smear examinations, and a reduction in the prescription of antibiotics for CAD, thereby markedly reducing management costs [14, 15]. In Jordan, Abu Rumman et al. found that the PAL approach decreased the number of drugs prescribed per patient by 12.2 % but increased the prescription of inhalers by 155 % [15]. Similarly, in Syria, Me’emary et al. showed a 14.8 % decrease in the number of drugs prescribed per patient and a decrease of 27.4 % in the proportion of patients who received antibiotic prescriptions [14].

Despite evidence for efficacy, the PAL strategy has not been universally adopted in most developing countries, including Malawi. This may be a result of poor acceptance and lack of political will by the national health authorities, or due to concerns regarding the feasibility of strategy implementation at primary health care facilities which are managed by low-level and middle-level health care workers.

The PAL strategy was designed for use in district hospitals. However, there is some evidence that the approach can be implemented at primary health care levels [13]. The studies in Syria and Jordan, as stipulated above, used qualified medical doctors to implement the PAL strategy. However, it is not clear whether it would be feasible to implement the PAL strategy in primary health care facilities staffed by low-level or middle-level health cadres such as clinical officers, medical assistants and nurses. Nonetheless, similarly adapted PAL approaches Practical Approach to Lung Health in South Africa (PALSA) and Practical Approach to Lung Health in Malawi (PALM) have been implemented in South Africa [16, 17] and Malawi [18], respectively, using low level cadres. However, these studies were primarily interested in assessing the impact of outreach training on the recruitment and retention of staff.

In addition to the feasibility challenges of implementing the PAL strategy in developing countries, access to health services in general, and in particular CAD and TB services, remains a challenge. These challenges are due to both, health system and patient barriers [19]. Health system barriers include inadequately qualified health staff with insufficient training and a lack of equipment and treatments.

Optimal utilisation of the available services is further restricted by patient barriers such as poverty [20], stigma, disability [21], low awareness levels for CAD and TB as well as geographical barriers. These barriers encourage patients to seek care from informal health providers such as traditional healers and grocery shop owners [22]. These informal health providers, in general, lack the knowledge and skills needed to provide appropriate health care information, treatment and referrals to health care centres. As a result, poor people accessing care from informal health providers often experience delays in treatment, waste time and resources, and worse still, experience poor health outcomes [23].

In view of these patient barriers, strengthening community-based structures could not only improve a community’s knowledge on CAD and TB but also service utilisation. Engagement of informal health providers in delivery of health interventions to promote service access is well documented. In Kenya, storekeepers have been used to recognise and treat uncomplicated malaria in children and to refer to health services when needed [6, 7]. In South Africa, storekeepers provided successful supervision of TB treatment – directly observed treatment (DOT) [24, 25]. A previous study in Malawi, conducted by the REACH Trust, demonstrated that storekeepers could promote TB case-finding [26] and further work by the REACH Trust demonstrated that informal providers can be engaged in the provision of integrated TB and HIV services at the community level. We found that these interventions led to improvements in HIV-testing by 61 %, anti-retroviral therapy (ART) access rates by 34 % and diagnostic uptake rates for presumptive TB cases by 15 % (manuscript in preparation).

### Aim

The proposed trial aims to determine the effect of Informal Health Provider and Practical Approach to Lung Health interventions on the detection and management of CAD and TB at primary care level in Malawi.

## Methods - trial design

A cluster level randomised controlled open trial with three arms.

### Trial sites

The trial sites will be in the districts of Dowa and Ntchisi, in the central region of Malawi. A total of 27 primary health centres in the districts will be involved in the trial. Each cluster will include a primary health centre together with the surrounding catchment area. To reduce contamination between clusters there will be a 3-km buffer zone between each cluster. The health facilities have been selected based upon their size according to the number of health workers and type of service offered. Those with in-patient services have been excluded. There are 11 such health centres in Ntchisi and 16 in Dowa district. Private not-for-profit health facilities, which run under the Christian Health Association of Malawi (CHAM), have been included.

### Selection criteria

#### Inclusion criteria for health facilities

1. Health facilities that offer out-patient services only
2. Run by low-level and middle-level health care workers

#### Exclusion criteria

1. Health facilities offering in-patient services
2. Health facilities run by qualified medical doctors

### Sources of recruitment

A community engagement exercise will be carried out to seek community leader and local community support for the trial. Participants selected for participation in the baseline or follow up household surveys will be visited at their homes and invited to participate.

#### Inclusion criteria for individual participants

All consenting individuals aged 15 and above will be eligible to participate.

#### Exclusion criteria

1. Individuals below the age of 15 years.
  According to TB control guidelines those below the age of 15 years are less likely to expectorate sputa for TB screening and they fall into a paediatric group
2. Refusal to participate in the survey will also be an exclusion criterion
3. Visiting members of the household

### Allocation of interventions

Each cluster (health centre with the surrounding catchment area) that has agreed to participate will be randomly allocated to one of the three trial arms using a computer-generated randomisation schedule with stratification by size of cluster. This randomisation will be performed by the trial statistician using dummy codes ‘A’ ‘B’ and ‘C’ to represent intervention and control groups; to ensure the statistician remains blinded, the identity (allocation) of ‘A’ ‘B’ and ‘C’ will be determined by a person independent of the study.

#### The interventions

- *Intervention arm 1* – Health centres implementing the PAL intervention. No informal provider intervention.
- *Intervention arm 2 –* Health centres implementing the PAL intervention. Informal provider intervention.
- *Control arm –* No intervention at health centre or informal provider level. Routine standard care will be provided.

#### Detail of the health centre-based PAL intervention in arms 1 and 2

The intervention at health centres will involve training medical assistants and nurses on the PAL guide-lines and use of spirometry for the diagnosis and assessment of airflow limitation in patients presenting with chronic cough. The trial will test the feasibility of implementing PAL strategy and integrating TB screening and CAD at the lowest level in the health system in Malawi. Lessons learnt are expected to influence policy and practice countrywide.

Other indicators to measure impact of the trial will include: occurrence and frequency of hospitalisations due to exacerbations, time lapse between symptom development and diagnosis. For TB patients, changes in this diagnostic delay may be a proxy indicator of health outcomes.

Training of health care providers will include:

1. Training of health centre staff on identifying and managing TB and CADs from amongst those with chronic cough.
2. Introducing the use of standardised registers for patients presenting with chronic cough.
3. Introducing the syndromic approach and spirometry for the diagnosis of airway diseases and TB among patients with chronic cough.
4. Introduction and uninterrupted use of inhalers (bronchodilators and corticosteroids) for patients diagnosed with asthma.
5. Introduction and uninterrupted availability and use of inhalers (bronchodilators) for patients diagnosed with COPD.

The aim is that after training the medical assistants and nurses in the intervention health centres will competently carry out the following tasks:

1. Screen and identify COPD, bronchiectasis, TB and asthma cases among patients presenting with chronic cough to primary health care facilities.
2. Use appropriate screening and diagnostic tools and specimens for the diagnosis of COPD, bronchiectasis, TB and asthma.
3. Make appropriate referral of patients to, and receive corresponding feedback from, secondary referral health facilities in the districts.
4. Perform accurate recording and reporting of all cases using tools for the PAL guidelines.
5. Provide key health messages about TB and CAD as part of the routine health talks.

#### Detail of the informal health provider intervention in arm 2

The intervention directed at the community level in intervention arm 2 will aim to build the capacity of informal health providers (IHPs) to recognise, counsel and refer potential TB and CAD patients in their communities.

Mapping of IHPs in the clusters allocated to intervention arm 2 will be done after randomisation. A qualitative study going on in the neighbouring district of Kasungu to explore how the communities understand IHPs by type and role will inform the trial to use the right definitions of informal health providers. During the mapping exercise distribution of IHPs will be determined prior to selection and training them in order to be within the buffer zones.

This intervention will include:

1. Training of informal health providers in the recognition, counselling and referral of patients with chronic/persistent cough to the primary health facility.
2. Training of informal health providers in prevention of COPD and including smoking cessation.
3. Training informal health providers to support patients with airway diseases so that they adhere to both drug and non-drug therapies and compliance to appointments.
4. Training of local health centre staff and health surveillance assistants to give supervisory support to informal health provider activities.
5. Establishment of referral mechanisms between the community and the health system.

Thus, once trained informal care providers will be required to routinely carry out one or more of the following tasks according to their capacity and prevailing circumstances as part of their daily activities:

1. Recognise selected presentations of chronic cough and potential TB cases among eligible persons, including persons with disability, in their communities.
2. Communicate effectively with patients and members of the public to i) encourage and support positive health-seeking behaviour, ii) provide key health messages about COPD, bronchiectasis, TB and asthma among chronic respiratory diseases and iii) provide reliable information on the available services for these diseases at the primary health care facilities in the area.
3. Advise on appropriate screening and diagnostic specimens for TB diagnosis (e.g. sputum).
4. Recommend and provide appropriate information about COPD, bronchiectasis, TB and asthma to encourage positive health care-seeking behaviour and adherence to specific disease risk reduction measures.
5. Refer to, and receive feedback from local public health services at primary health care level, for all identified cases with CADs and suspected TB cases.
6. Competently record and report on all cases referred to primary health care and activities done in the community.

### Outcome assessments

#### Primary outcome measures

1. Proportion of the population with a chronic cough who have a diagnosis of TB or airway disease(s) recorded in their health passports.

This has been selected as the primary outcome since this is the most clinically relevant outcome effect measurable immediately downstream of the intervention. This intervention is measurable at the community level, thus requiring a community-based survey to collect outcome data.

#### Secondary outcome measures

1. Proportion of the population with a chronic cough on salbutamol/corticosteroid inhaler indicated in their health passports.
2. Proportion of the population with a chronic cough with a diagnosis of TB or airway disease among patients with chronic cough attending primary health care recorded in patient registers at intervention facilities.
3. Proportion of people with disabilities with a diagnosis of TB or airway disease recorded in their health passports in all arms of the study.

In order that diagnoses of CAD, including asthma and TB are made with a high level of accuracy, appropriate merit-based training of spirometry technicians will be done by a competent (PATS/ERS/ATS) certified trainer. Spirometry technicians will further undergo refresher training during the trial. Diagnosis of TB will be based on the routine practice of TB microscopy and chest X-rays (CXRs). In some instances the Gene Expert test will be performed according to an available standard algorithm. We will consider to undertake a systematic investigation of the accuracy of diagnoses made in a subset of patients by checking results, clinical notes in patient health passports, and CXR’s, along with a thorough clinical review.

Limitations for diagnosis include lack of sputum culture for TB diagnosis and computed tomography (CT) scan for conditions like bronchiectasis. Unfortunately sputum culture and CT scans are beyond the funding scope of the study.

### Case report forms

Electronic case report forms (CRFs) programmed onto smartphones using Open Data Kit (ODK) will be used to make the large number of CRFs manageable and provide real-time data entry, internal validity and consistency checks. CRFs will be treated as confidential documents and held and backed up on two secure servers. Data will be anonymised prior to analysis.

### Potential risks to the safety of the trial participants

Trial participants will be recruited from populations living in poor conditions in Malawi who live with relatively high day-to-day risks. The interventions are particularly low-risk and targeted at improving access to medical care for those at need. Overall we anticipate that participation in the trial will reduce risks to participants.

### Adverse event reporting

An adverse event (AE) is any unfavourable and unintended sign, symptom, syndrome or illness that develops or worsens during the period of observation in the study. A Serious Adverse Event (SAE) is an AE following the intervention that results in a) death, b) a life-threatening AE, c) hospitalisation or prolongation of an existing hospitalisation, d) disability or incapacity, e) congenital anomaly in the offspring of a participant.

The interventions we will be using in this trial are particularly low-risk interventions that offer potential safety benefits (e.g. improved access to care). Nevertheless, we will collect data about AEs. Data about AEs that are not serious will be collected with the population-based survey at the end of the trial. Participating health centres will be asked to report SAEs in relation to the interventions immediately to the trial coordinating centre. The trial coordinating centre will collect details about the SAE using a pro-forma in accordance with a specific standard operating procedure (SOP). This information will then be passed immediately to Hastings Banda who will conduct a causality assessment (not related/improbable, possible, probable, definite), assess seriousness and expectedness, take any appropriate medical action and inform the College of Medicine Research Ethics Committee (COMREC) and the Liverpool School of Tropical Medicine Research Ethics Committee (LSTM REC) of any events deemed related to the trial intervention within 7 days of knowledge of the event. All other SAEs will be reported as part of an annual report to COMREC and LSTM REC. All SAEs that relate to the interventions will be followed to resolution.

### Sample size and sampling approach

The primary objective of this study is to measure the impact of the interventions on the proportion of chronic cough patients with a diagnosis of CAD or TB in their health passport. The preliminary sample size considerations set out below are based solely on expert opinion; these assumptions and estimations for the follow-up impact study will be revisited in light of the data collected during the baseline survey.

#### Baseline survey

Data on CAD is not routinely collected in the study districts, so to inform the final sample size estimation a baseline survey will be conducted, the purpose of which will be to estimate the proportion of individuals over the age of 15 years in each cluster with (a) evidence of chronic cough-/- wheeze, and (b) a diagnosis in their health passport.

For this baseline survey, it will be assumed that for the study population:

(i) the mean household size in the study area will be 4.6
(ii) the proportion of individuals over the age of 15 years will be 0.52 (52 %)
(iii) so the average number of individuals per household over the age of 15 years will be 4.6 × 0.52 = 2.4

As described in detail in table 1, a 3-stage sampling procedure will be used:

- Stage 1: a random sample of 27 clusters (health centres with surrounding catchment areas) will be selected and 9 of these randomly allocated to each study arm
- Stage 2: 30 villages will be randomly selected from each of these 27 clusters based on sampling probabilities proportional to village population again using Google Earth Pro (Google Inc., Mountain View, CA, USA) to identify villages and to estimate their sizes (total 810 villages: 270 per study arm)
- Stage 3: a simple random sampling procedure will be applied to select 7 households from each selected village (total 5,670 households: 1,890 per study arm)

All eligible participants in each selected household will be interviewed, giving a total sample of 5,670 × 2.4 = 13,608 individuals (4,536 per study arm); 10 % of these individuals (1,360 in total: 453 per study arm) will be expected to show evidence of chronic cough-/-wheeze.

The precision figures quoted below for this baseline survey use the approximate formula for a 95 % confidence interval ‘1.96 × standard error of prevalence estimate’. In the actual analysis, exact binomial confidence intervals will be computed, but these are asymmetric so unsuitable for presenting a simple indication of the precision levels that will be achieved.

Assuming a prevalence of 10 %, a sample of 4,536 individuals will provide an estimate of the true prevalence of chronic wheeze-/-cough in each individual study arm with a precision of ±0.87 %. Ignoring clustering effects, the 13,608 individuals in all 3 study arms combined will provide a precision of ±0.50 %; adding a design effect size adjustment of 2 to allow for clustering effects, the combined sample will have a precision of ±0.71.

Assuming that 5 % of individuals who demonstrate symptoms of chronic cough-/-wheeze will have a diagnosis of CAD or TB in their health passport, a sample of 453 individuals will provide an estimate of the true health burden prevalence in each individual study arm with a precision of ±2.00. Ignoring clustering effects, the 1360 individuals in all 3 study arms combined will provide a precision of ±1.17 %; adding a design effect size adjustment of 2 to allow for clustering effects, the combined sample will have a precision of ±1.64 %.

(N.B. The effects of clustering within households, within villages and within health centre catchment areas will all be fully adjusted for in the statistical analysis using multi-level modelling methods). A summary of sampling steps and estimated numbers for the baseline survey is shown in Additional file 1: BANDA Additional file 1.pdf, 235 K.

#### Estimation of impact of PAL alone and PAL + IHP interventions versus control

The calculations presented below use an alpha level of 2.5 % rather than the conventional 5 % to allow for 2 primary comparisons (PAL + IHP versus control and PAL alone versus control).

Exactly the same 3-stage sampling procedure will used as for the baseline survey, except that now 14 households will be selected from each village (total 11,340 households: 3,780 per study arm). As before, all eligible participants in each selected household will be interviewed, giving a total sample of 11,340 × 2.4 = 27,216 individuals (9,072 per study arm); 10 % of these individuals (2,721 in total: 907 per study arm) will be expected to show evidence of chronic cough-/wheeze.

The available evidence indicates that approximately 5 % of symptomatic individuals in the control group post-intervention will have a diagnosis in their health passport. The PAL + IHP intervention will be considered clinically important if it increases the proportion of symptomatic individuals with a registered diagnosis from 5 to 20 %.

With 9 clusters per group and 907 symptomatic individuals per study arm (100 per cluster), this study will have 80 % power to detect a clinically important difference between the PAL + IHP and control groups provided the BCV (between-clusters CV (coefficient of variation)) does not exceed 62.9 %, and will have 90 % power if the BCV does not exceed 54 %.

BCV measures the extent to which the proportion of symptomatic individuals with a registered diagnosis varies between the clusters, and expresses this as a percentage of the average proportion of symptomatic individuals with a registered diagnosis across all clusters combined. In most community interventions, BCV is usually less than or equal 25 % [27] so, even at worst, this element of the study will have over 90 % power.

The PAL alone intervention will be considered clinically important if it increases the proportion of symptomatic individuals with a registered diagnosis from 5 to 10 %. With 9 clusters per study arm and 100 symptomatic individuals per cluster, the study will have 80 % power to detect a clinically important difference between the PAL alone and control groups provided the BCV does not exceed 24.1 %, and 90 % power if the BCV does not exceed 13.7 % (so the actual power of this element of the study is expected to be at, or close to, 80 %). A summary of sampling steps and estimated numbers for the post-intervention survey is shown in Additional file 2: BANDA Additional file 2.pdf,233 K.

#### Data collection and management

The baseline survey will be conducted prior to randomisation and prior to intervention implementation. The follow-up survey will be conducted 1 year after intervention implementation. Data will also be gathered from health centres 1 year after the interventions have been implemented.

Measures will be taken to ensure quality and accuracy of information, which will be collected and reported; these will include: firstly, thorough training of health workers – the training content will include proper recording of diagnoses based on knowledge of presenting symptoms and signs of each condition and the differential diagnoses. In addition health care workers will also be taught about spirometry results so that they acquire understanding to help with them in interpreting spirometry results to make accurate diagnosis. Secondly, the trial clinical supervisor will routinely undertake monitoring of practice in the intervention health facilities to ensure that standards are being followed regarding practice, recording and reporting. Patient health passports, PAL registers at facilities and spirometry records will be assessed during the visits.

The data collected from health registers as digital images will be scanned into an electronic database using Teleform software (Cardiff Software, Sunnyvale, CA, USA) or, if this proves to be technically difficult; the scanned images will be double-entered by trained data entry clerks into an Microsoft Access for windows or Epi-Info 6.04 database, a free software developed by the Center for Disease Control of Atlanta in the United States of America.

All other quantitative data will be collected on pre-prepared and validated pro-formas by trained researchers. All forms completed on any given day will be checked visually at the end of the day and any data queries identified (including missing entries) will be checked with theresearchers. The data will then be double-entered onto a pre-prepared Access or Epi-Info computer database, and the original pro-formas will be stored in locked filing cabinets in a secure area within the research centre. This will serve as a data security measure and also to protect confidentiality. The computer server holding the Epi-Info databases will be backed-up daily to a second server held in an area physically separate from the primary server; a secondary back-up will also be made onto a portable disk-drive, which will be held in a locked cabinet for safety and security.

All principal investigators will be given access to the final cleaned data sets. Data will be anonymised to maintain confidentiality.

### Statistical analysis plan

The characteristics of the individuals recruited into study surveys will be summarised using means, median or proportions, as appropriate. All summary statistics will be accompanied by their standard deviations or 95 % confidence intervals, again as appropriate. Missing or invalid observations detected during this analysis will be referred back to the field-based researchers for clarification.

Effect sizes for the study outcome measures will be summarised using incidence rate ratios with their 95 % confidence intervals. Negative binomial and/or Poisson regression methods will be used to adjust these effect sizes for potential influencing/confounding factors; the influence of these factors on the effect size incidence rate ratio estimate will be reported for each factor individually and then for all factors combined. Multiple imputation methods will be used to accommodate any missing observations that could not be clarified by the field researchers.

The analyses will be conducted under the supervision and advice of Dr. Brian Faragher, a senior lecturer in Medical Statistics at Liverpool School of Tropical Medicine.

#### Interim analyses and stopping rules

Data collection for this trial will be conducted at baseline and at the end of the trial. There will, therefore, be no opportunity for an interim analysis to inform stopping the trial early. We consider this to be acceptable given the low-risk nature of the trial.

### Ethical aspects

#### Ethics committee approvals

The protocol has been reviewed and fully approved by the COMREC in Malawi. Ethical approval has also been obtained from the Liverpool School of Tropical Medicine.

#### Protocol amendments

Any significant amendment to the protocol will require agreement of the TSC and approval of both COMREC and LSTM REC. Information regarding protocol amendments will be disseminated to relevant parties including investigators, field workers, health centres, IHPs and participants as appropriate.

#### Informed consent form and information sheets

Prior to conducting each and every interview, an information sheet will be used to fully inform the potential participant. Prior to completing any interviews or collecting any study-related data, a consent form will be completed and signed. This process with be adapted for any potential participants who are unable to read such that the information sheet will be read out and the consent form completed with assistance from the field team. A mark witnessed by someone independent to the study will be accepted where participants are unable to sign.

Informed consent will be obtained by the following team from the REACH Trust:

1. Hastings TR Banda; Principal Investigator, Dip Clin Med (Malawi), MCommH (Liv), UK
2. Grace Bongololo Mbera; Social Scientist, BA, MA (Mw)
3. Kassim Kwalamasa, Social Scientist, BA (Mw)
4. Martina Mchenga, Health Economist, BA, MA (Mw)
5. Blessings Chisunkha, Data Coordinator, BSc (Mw)
6. Brian Ngwira, Clinical Supervisor, Dip Clin Med (Mw), BSc Med (Mental Health) (Mw).

#### Consideration of potential risks to participating health centres and their catchment populations

The interventions being tested in this cluster level randomised trial are applied at a community or health centre level. At a community level, this is a signposting system and does not involve any treatments. At the health centre level we will be implementing an advanced care package that only contains components that would be considered standard good practice. At intervention health centres, each individual will, therefore, be managed with standard care and the additional diagnostic capabilities and treatments. Training will be given on the management of CAD and TB including managing any side effects of inhalers. As neither of the interventions are medications we have not included specific criteria for the modification or discontinuation of interventions. Individual treatments that have been initiated at the health centres can be discontinued by the patients at any time. There are no restrictions on the concomitant care that participants may receive during the trial.

If patients receive a diagnosis of CAD they may be given either salbutamol and/or corticosteroid inhalers, these have minimal side effects. Salbutamol inhalers may lead to a transient increase in heart rate, decreased plasma potassium concentration and decreased diastolic pressure. Side effects of corticosteroid inhalers include oesophageal candidiasis, dysphonia, wheezing and cough. Prolonged use of inhaled corticosteroids may increase the risk of developing TB in patients who are being treated for COPD [27]. Although the drugs used in this study are routinely used in health facilities in Malawi, the study will closely monitor and document any reported side effects and where possible the frequency of intake of these drugs may be reduced. Health facility staff will manage the side effects and refer to the district hospital if appropriate. Furthermore, adequate training in the use of inhalers and corticosteroids and side effects recognition will be given to minimise the occurrence of these side effects. Training on use of spirometers for diagnosing airflow limitation will also be given. This training will be facilitated by a chest specialist (Dr. Kevin Mortimer) who is part of this study.

### Trial management

#### Trial protocol review and registration

The protocol was submitted for review and has been registered with The Pan African Clinical Trial Registry (www.pactr.org) database and will be published in an open access format. Registration data set details are in Additional file 3: BANDA pdf, 231 K.

#### Arrangements for day-to-day management

A full time on-site qualified and experienced clinical officer (Hastings Banda) has been appointed and will have responsibility for day-to-day management issues, supervision of field workers and data manager, protocol compliance, security of the randomisation process, recruitment, data management, problem identification and resolution, distribution and maintenance of trial materials, budget control and production of annual progress reports. Hastings Banda will be supported by the REACH Trust, a trial management group and a supervisory group (Moffat Nyirenda, Kevin Mortimer, Bertie Squire, and Rachael Thomson).

The Trial Management Group (TMG) led by the clinical officer will be established to manage the trial on a day-to-day basis. The TMG will meet at least monthly and will monitor trial conduct, progress, adherence to the protocol and SOPs, CRF completion, accuracy and completeness of data collection, data validity and, where necessary, act to safeguard trial participants and quality standards. The TMG will receive logistic and infrastructural clinical trials support from the Liverpool School of Tropical Medicine Tropical Clinical Trials Unit.

#### Research governance arrangements

Trial oversight committees will be established including the TMG and the trial steering committee (TSC). LSTM as trial sponsor will perform an initial start-up and then annual Good Clinical Practice (GCP) compliance visits to ensure and document complete compliance. All trial staff and investigators will protect the rights of participants to privacy and informed consent. Internal quality control monitoring will be conducted 3-monthly to ensure understanding of protocol and SOPs, protocol and GCP compliance, conduct source document (health passports) verification and confirm that all participating households have given written informed consent. The internal quality control monitoring will be conducted by the investigators and the TSC. This process is independent of the trial sponsor. Participation in the trial involves low risk interventions and procedures that are not expected to increase the risk of SAE above the normal baseline for poor people in Malawi. All SAEs will be reported to the TSC and via onward reports to COMREC and LSTM REC. Trial participants and trial staff will be covered by LSTM indemnity and insurance. The principal investigators will maintain all records and documents regarding the conduct of the study and retain these for at least 7 years or for longer if required. The Trial Master File and trial documents shall be finally archived at secure archive facilities at LSTM or the REACH Trust.

#### Data Monitoring Committee

As discussed by the TSC, a Data Monitoring Committee is not considered necessary given the low risk nature of the trial interventions and given the lack of interim data analysis opportunities. Any data monitoring that may be required will be overseen by the TSC.

#### Trial Steering Committee

A TSC has been established to provide overall supervision of the trial and is chaired by a member from among independent members who are in majority. The TSC meets face-to-face prior to trial initiation to agree the trial protocol and terms of reference and will convene at least annually henceforth through conference calls and one further face-to-face meeting if needed. The principal investigator in association with the chair will take responsibility for calling and organising TSC meetings.

### Timeline

The project is planned to run from January 2014 to December 2016. Interventions are planned to be implemented from March 2014 to February 2015. The final results are expected in December 2016.

### Publication and dissemination of results

The trial findings will be presented at international conferences and published in peer-reviewed journals in an open access format. Results will be communicated to the participating health centres. The district health management team and the district executive committee will be briefed regarding the trial results.

## Discussion

The trial will undertake two cross-sectional household surveys: one at the beginning of the trial, prior to randomisation of clusters, and the second after the interventions have been in place for a year. Although the intervention is health centre-based to improve quality of service the primary outcome measure will be assessed in the communities. Trial data, therefore, will have several sources including cross-sectional household surveys, clinic registers, and patient health passports. Monitoring and supervision tools will be developed to support the completeness and accuracy of these data. A subset of patients will be selected to assess quality and accuracy of diagnosis and patient management.

### Trial status

This trial has approval from COMREC as well as from LSTM REC. All health centres have been selected. The trial has been registered with the Pan African Clinical Trials Registry.

## Additional files

Additional file 1:
**Baseline sampling steps and summary of estimates.** (DOCX 18 kb) Additional file 2:
**Post-intervention sampling steps and summary estimates.** (DOCX 19 kb) Additional file 3:
**Trial registration details.** (DOCX 21 kb)

## Abbreviations

AE: adverse events
ART: anti-retroviral therapy
BCV: between-clusters CV
CAD: chronic airways disease
CHAM: Christian Health Association of Malawi
COMREC: College of Medicine Research Ethics Committee
COPD: chronic obstructive pulmonary disease
CRF: case report forms
CT: computed tomography
CV: coefficient of variation
CXR: chest X-ray
DOT: directly observed treatment
GCP: Good Clinical Practice
HIV: human immuno-deficiency virus
IHPs: informal health providers
IUATLD: International Union Against Tuberculosis and Lung Disease
LHL: The Norwegian Health and Lung Patients Organisation
LSTM: Liverpool School of Tropical Medicine
LSTM REC: Liverpool School of Tropical Medicine Research Ethics Committee
NCCRD: non-communicable chronic respiratory diseases
NCD: non-communicable diseases
NORAD: Norwegian Government
ODK: Open Data Kit
OPD: out-patient department
PAL: Practical Approach to Lung Health
PALM: Practical Approach to Lung Health in Malawi
PALSA: Practical Approach to Lung Health in South Africa
PHC: primary health care
REACH: Research for Equity and Community Health
REC: Research Ethics Committee
SAE: serious adverse events
SOP: standard operating procedure
SSA: sub-Saharan Africa
TB: tuberculosis
TMC: Trial Management Committee
TMG: Trial Management Group
TSC: Trial Steering Committee
WHO: World Health Organisation
