# Supplementary material for: Informal Health Provider and Practical Approach to Lung Health interventions to improve the detection of chronic airways disease and tuberculosis at primary care level in Malawi: study protocol for a randomised controlled trial
Source: Trials. 2015 Dec 17;16:576. doi: 10.1186/s13063-015-1068-4 (PMC4683704; doi:10.1186/s13063-015-1068-4)
Supplement: Additional file 1: — Baseline sampling steps and summary of estimates. (DOCX 18 kb) [file 13063_2015_1068_MOESM1_ESM.docx]

[Ad d it ion al f ile 1: Ad d it ion al file 1.p df : Baselin e Samp lin g step s an d su mmary o f est imates](https://lstmed.sharepoint.com/sites/RespiratoryGroup/HAP/CAPS/1.%20Administration/Hastings/Old%20additional%20files/Additional%20file%201.pdf)

| **Baseline Survey – Summary of sampling steps & estimated numbers** | | | | | |
| --- | --- | --- | --- | --- | --- |
|  | | **Study arms (number of people)** | | | |
| **Row** | **Explanation** | **PAL** | **PAL plus IHP** | **Control** | **Total (All arms)** |
| **A** | Number of clusters (health centres with surrounding catchment areas) | 9 | 9 | 9 | 27 |
| **B** | Random selection of 30 villages per cluster from Google Earth | 30 | 30 | 30 |  |
| **C** | Number of villages selected (C = A x B) | 270 | 270 | 270 | 810 |
| **D** | Random selection of 7 households within each village | 7 | 7 | 7 |  |
| **E** | Number of households selected (E = C x D) | 1890 | 1890 | 1890 | 5,670 |
| **F** | Estimated number of adults per household | 2.4 | 2.4 | 2.4 |  |
| **G** | Expected number of individuals to be interviewed  (G = E x F) | 4536 | 4536 | 4536 | 13,608 |
| **Baseline estimates of disease burden** | | | | | |
| **H** | Estimated percentage reporting chronic cough | 10% | 10% | 10% |  |
| **I** | Expected number of individuals with chronic cough  (H=G x H) | 453 | 453 | 453 | 1,359 |
| **J** | Estimated percentage with CAD +/or TB diagnosis in health passport at baseline | 5% | 5% | 5% |  |
| **K** | Expected number of patients with CAD +/or TB diagnosis in health passport at baseline  (K = I x J) | 23 | 23 | 23 | 69 |
|  | | | | | |
