# Supplementary material for: Informal Health Provider and Practical Approach to Lung Health interventions to improve the detection of chronic airways disease and tuberculosis at primary care level in Malawi: study protocol for a randomised controlled trial
Source: Trials. 2015 Dec 17;16:576. doi: 10.1186/s13063-015-1068-4 (PMC4683704; doi:10.1186/s13063-015-1068-4)
Supplement: Additional file 2: — Post-intervention sampling steps and summary estimates. (DOCX 19 kb) [file 13063_2015_1068_MOESM2_ESM.docx]

[Ad d it ion al f ile 2: Add ition al f ile 2.p df : Post- inter vent ion Samp lin g step s an d su mmar y](https://lstmed.sharepoint.com/sites/RespiratoryGroup/HAP/CAPS/1.%20Administration/Hastings/Old%20additional%20files/Additional%20file%202.pdf)

[est imates](https://lstmed.sharepoint.com/sites/RespiratoryGroup/HAP/CAPS/1.%20Administration/Hastings/Old%20additional%20files/Additional%20file%202.pdf)

**s**

| **Post-intervention Survey – Summary of samp** | | | **ling steps** |  | **& estimate** | | **d number** |  | ***** |
| --- | --- | --- | --- | --- | --- | --- | --- | --- | --- |
|  |  |  | **Study arms (number** | | |  | **of people** |  | **)** |
| **Row** | **Explanation** |  | **PAL** |  | **PAL plus IHP** |  | **Control** |  | **Total (All arms)** |
| **A** | Number of clusters (health centres with surrounding catchment areas) |  | 9 |  | 9 |  | 9 |  | 27 |
| **B** | Random selection of 30 villages per cluster from Google Earth |  | 30 |  | 30 |  | 30 |  |  |
|  |  |  |  |  |  |  |  |  |  |
| **C** | Number of villages selected (C = A x B) |  | 270 |  | 270 |  | 270 |  | 810 |
| **D** | Random selection of 14 households within each village |  | 14 |  | 14 |  | 14 |  |  |
| **E** | Number of households selected (E = C x D) |  | 3780 |  | 3780 |  | 3780 |  | 11,340 |
| **F** | Estimated number of adults per household |  | 2.4 |  | 2.4 |  | 2.4 |  |  |
| **G** | Expected number of individuals to be interviewed  (G = E x F) |  | 9072 |  | 9072 |  | 9072 |  | 27,216 |
|  |  |  |  |  |  |  |  |  |  |
| **H** | Estimated percentage with CAD +/or TB diagnosis in health passport at study end |  | 10% |  | 20% |  | 5% |  |  |
| **I** | Expected number of patients with CAD +/or TB diagnosis in health passport at study end  (I = G x H) |  | 907 |  | 1814 |  | 454 |  | 3175 |
