# Supplementary material for: Informal Health Provider and Practical Approach to Lung Health interventions to improve the detection of chronic airways disease and tuberculosis at primary care level in Malawi: study protocol for a randomised controlled trial
Source: Trials. 2015 Dec 17;16:576. doi: 10.1186/s13063-015-1068-4 (PMC4683704; doi:10.1186/s13063-015-1068-4)
Supplement: Additional file 3: — Trial registration details. (DOCX 21 kb) [file 13063_2015_1068_MOESM3_ESM.docx]

[Ad d it ion al f ile 3: Ad d it ion al file 3.p df : Trial Registrat ion d etails](https://lstmed.sharepoint.com/sites/RespiratoryGroup/HAP/CAPS/1.%20Administration/Hastings/Additional%20file%203.pdf)

| **Data category** | **Information** |
| --- | --- |
| Primary registry and trial identifying number | Pan African Clinical Trial Registry (www.pactr.org). The unique identification number for the registry is PACTR201411000910192 |
| Date of registration in primary registry | November 21 2014 |
| Secondary identifying numbers | N/A |
| Source(s) of monetary or material support | Norwegian Heart and Lung Patient Association (LHL) International Tuberculosis Foundation (LHL International) and the Norwegian Government (NORAD) |
| Primary sponsor | Liverpool School of Tropical Medicine Pembroke Place  Liverpool L3 5QA UK  Tel: +44(0)151 705 3100  E-mail: [lst minfo@liv.ac.uk](mailto:lstminfo@liv.ac.uk) |
| Secondary sponsor(s) | N/A |
| Contact for public queries | The REACH Trust  P.O. Box 1597, Lilongwe, Malawi  Tel: +265 (0) 1 753 260/261  E-mail: [info@rea cht ru st .org](mailto:info@reachtrust.org) |
| Contact for scientific queries | Hastings T. Banda The REACH Trust  P.O. Box 1597, Lilongwe, Malawi  Tel: +265 (0) 1 753 260/261  E-mail: [h ast in gs@reacht ru st .org](mailto:hastings@reachtrust.org) |
| Public title | Informal Health Provider and Practical Approach to Lung Health interventions to improve the diagnosis and treatment of chronic cough in Malawi |
| Scientific title | Informal Health Provider and Practical Approach to Lung Health interventions to improve the detection of chronic |

| **Data category** | **Information** |
| --- | --- |
|  | airways disease and tuberculosis at primary care level in Malawi: A cluster level randomized controlled trial |
| Countries of recruitment | Malawi |
| Health condition(s) or problem(s) studied | Chronic cough |
| Intervention(s) | Practical Approach to Lung Health strategy intervention +/- training of informal healthcare providers within the community. |
| Key inclusion and exclusion criteria | **Inclusion Criteria**  All consenting individuals aged 15 and above  **Exclusion Criteria**   1. Individuals below the age of 15 years 2. Refusal to participate 3. Visiting members of the household |
| Study type | Cluster level randomised controlled open trial with three arms. |
| Date of first enrolment | To be confirmed |
| Target sample size | 27 000 |
| Recruitment status | All Health centres have been selected. |
| Primary outcome(s) | Proportion of the population with a chronic cough who have a diagnosis of TB or airway disease(s) recorded in their health passports. |
| Key secondary outcomes | 1. Proportion of the population with a chronic cough on salbutamol / corticosteroid inhaler indicated in their health passports. 2. Proportion of the population with a chronic cough with a diagnosis of TB or airway disease among patients with chronic cough attending primary health care recorded in patient registers at intervention facilities. 3. Proportion of people with disabilities with a diagnosis of TB or airway disease recorded in their health passports in all arms of the study. |
